# Powder and Reconstituted Properties of Commercial Infant and Follow-On Formulas †

**DOI:** 10.3390/foods9010084

**Published:** 2020-01-13

**Authors:** Eoin G. Murphy, Nicolas E. Regost, Yrjö H. Roos, Mark A. Fenelon

**Affiliations:** 1 Teagasc Food Research Centre, Moorepark, P61 C996 Cork, Ireland; 2 Europe/North America Product Development & Global Research, H&H Group, 14 Rue du Vieux Faubourg, 59042 Lille, France; 3 School of Food and Nutritional Sciences, University College Cork, T12 K8AF Cork, Ireland

**Keywords:** infant formula, microstructure, emulsion quality

## Abstract

The physical properties of 15 commercially available infant formulas (IF) and follow-on (FO) formulas were analysed. Powders made with intact milk proteins were classified into two groups; Type I—homogenous mixtures of milk powder particles (*n* = 6); and Type II—heterogeneous mixtures of milk powder particles and tomahawk-shaped α-lactose monohydrate crystals (*n* = 6). Powders made using hydrolysed proteins were classified as Type III powders (*n* = 3). Type II powders exhibited similar flow characteristics to Type I powders despite having significantly (*p* < 0.05) smaller particle size, lower circularity, and greater elongation. Type III powders exhibited lowest particles size, highest surface free fat, and poorest flow properties (*p* < 0.05 for all). Upon reconstitution of powders (12.5% *w*/*w*), no significant difference (*p* < 0.05) in apparent viscosity was observed between Type I and II powders. Reconstituted Type III powders had relatively poor stability to separation compared to Type I and II powders, caused by large starch granules and/or poor emulsification by hydrolysed proteins. Overall, this study illustrated the range of physical behaviour and structures present in commercial IF powders. In particular, the effect of dry addition of lactose and the hydrolysis of protein were found to have major effects on physical properties.

## 1. Introduction

Infant formula (IF) powders are dehydrated emulsions consisting of protein, fat carbohydrate, vitamins, and minerals necessary to nourish infants in the absence of breast milk. As an infant grows and solid foods are introduced, complimentary follow-on (FO) formulas can be used as supplementary food. Most IF and FO powders are made with intact bovine proteins, however, specialised products also exist e.g., IF made with hydrolysed caseins and whey proteins for infants showing adverse reactions to standard formulations [1]. The U.S. Food and Drug Administration classify IF manufacturing processes into three categories: (1) Wet processing, (2) dry processing, or (3) a combination of wet and dry-processing. In wet processing, ingredients are hydrated in water to the desired composition and are subsequently spray-dried, as described by McCarthy et al. [2]. Dry processing involves mixing dried ingredients and is often employed in combination with spray-dried base powder; for example, Mullane et al. [3] described a process where a base powder containing fat and protein was manufactured and subsequently dry-blended with lactose, vitamins, and minerals to produce a final powder. 

Process-composition interactions during manufacture have large effects on the physical quality of dairy and IF powders [2,4,5,6,7]. For example, heat treatment causes denaturation of β-lactoglobulin (β-lg), the most abundant whey protein in bovine milk. Denaturation and subsequent aggregation with adjacent β-lg molecules and/or casein micelles causes viscosity increase during heat treatment [8,9]. Composition of IF wet mixes has been shown to be a key factor determining the onset and extent of these whey protein interactions and subsequent viscosity increases [10]. In contrast, hydrolysis of whey proteins in IF is a compositional consideration which can result in lower viscosity in wet-mixes [11].Viscosity, in turn, is a key parameter in determining initial powder particle size, which can also be manipulated by adjusting the level of agglomeration occurring during drying [12,13]. 

Large powder particle sizes generally increase flowability due to a reduction in the area of contact between particles during flow [14,15]. However, other factors can affect flowability, which in some cases can lead to a weak relationship between particle size and flowability. The surface composition of powder particles is especially important in dehydrated emulsions and high quantities of non-emulsified fat at the powder surface can reduce flowability [16,17]. Surface free fat is affected by dry matter concentration and degree of homogenization in concentrates prior to spray-drying, during which, the temperature profile within the dryer is also important [4,5]. Shape of particles can also affect flowability of powders, with more spherical powders having greater flowability [18]. In IF products where dry-blending of lactose occurs, large quantities of pyramidal or tomahawk shaped α-lactose monohydrate crystals will be present, which may reduce spherocity of particles, and thus, flowability of powders. 

Rehydration properties of powders are also influenced by manufacturing conditions. Wettability, the time it takes a given quantity of powder to sink below the surface of water at a certain temperature, improves with increasing particle size and is adversely affected by high surface free fat content [14,19,20]. The rate at which rehydrated IF powders destabilise, or “cream”, is related to the extent of emulsification of fat by protein prior to spray-drying [2]. Similarly, the discrete visible and insoluble particles, known as white flecks, which are occasionally present post-rehydration of IF powders, have been shown by Toikkanen et al. [21] to be linked to emulsion quality. Hydrolysis of whey proteins in IF can also be expected to affect emulsification and related properties such as creaming. Both positive and negative effects on emulsification have been reported with the exact impact being a function of degree of hydrolysis (DH), molecular weight, and amphiphilicity of peptides produced [22,23]; Kelly et al. [24] found that hydrolysis (DH = 12) of whey proteins in a model IF resulted in lower emulsion quality and higher creaming rates compared to formulations with intact whey protein. 

In recent years, there has been an increase in the publications dealing with IF manufacture [2,6,7,21,25,26,27,28]. Some publications [25,27,28] provided limited information regarding the physical properties of commercial IF and FO samples; however, it is difficult to discern usable information regarding the range of structures and physical properties present in commercial products from these studies. Such information would be a valuable resource for researchers and product developers striving to attain commercial product quality from novel processes and/or recipes. The current study was based on the hypotheses that significant differences exist in the physical characteristics of commercial IF and FO products and that investigating these differences may yield useful information relating to the effect of processing and composition on powder structure and quality. In particular, a strong emphasis was placed on the effects of dry-blending of lactose and hydrolysis of proteins in formulations. Therefore, the present study characterised the ranges of physical properties present in commercial products and related the observed differences to processing (e.g., 100% spray-dried vs. dry-blended) and compositional (e.g., intact vs. hydrolysed protein) factors during manufacture.

## 2. Materials and Methods

### 2.1. Commercial Infant Formula

A total of 15 IF powders were studied. Of these, 12 samples were purchased, and 3 samples were donated by H&H Group (Guangzhou, China). Table 1 shows the different types of formula studied. Whey-to-casein ratio was not specified in some of the commercial samples measured. Crystalline structure of the powders was determined using polarised light microscopy (Olympus Corporation, Tokyo, Japan) and powders were subdivided into three categories; I—a homogenous mix of milk powder particles; II—a heterogeneous mix of milk powder particles and crystalline particles; and III—IF with hydrolysed (comfort) proteins. Crystallisation under ambient conditions was also monitored; approximately 10 g of each powder was exposed to room conditions for 2 days and crystallisation behaviour was observed using light microscopy. 

### 2.2. Powder Properties

Particle size distribution was measured by a Mastersizer 3000 (Malvern Instruments Ltd., Malvern, UK). For this, 10 kPa air pressure was applied to the induction pipe, meaning the vacuum applied to the induction system was mostly responsible for powder induction. This low air pressure was necessary for agglomerated powders so as not to break the agglomerate structure. Powder particle size distributions were distributed normally and did not show any shoulders extending into larger particle size regions, such as is observed when non-agglomerated powders (e.g., lactose/milk protein concentrate) are not sufficiently dispersed. Refractive indices used for dispersant (air) and particles were 1 and 1.45, respectively [7]. Particle absorbance index was 0.1. Sauter mean diameter, D [3,2], which gives the diameter of a sphere with the same volume-to-surface-area ratio of the whole distribution was used as a measure of particle size. D (v, 0.1), which gives the diameter below which 10% of the distribution (by volume) lies, was used to quantify the number of fine particles in the distribution. The span of particles was calculated based on the following equation; Span = [D (v, 0.9)–D (v, 0.1)]/D (v, 0.5), where D (v, 0.9) and D (v, 0.5) represent the diameters below which 90% and 50% of the distribution lie, respectively.

Particle shape was measured by Morphologi G3 (Malvern Instruments Ltd., Malvern, UK). Powders were dispersed on to a microscope plate, using pressurised air. It was necessary to use high dispersion energy (as indicated by instrument software) to achieve adequate separation of particles. The air pressure used was 400 kPa which was applied to the sample dispersion for 10 milliseconds; a settling time of 1 min was then allowed for powder particles to disperse on the microscope plate. For Type I powders, 30 mm^3^ of powder was dispersed. It was observed for Type II powders that when using 30 mm^3^ of dispersed powder, there was over twice the amount of particles on the microscope plate which were not adequately separated; therefore, for Type II powders, the sample volume was reduced to 15 mm^3^. Surface free fat was determined as per GEA Niro method [29].

Bulk density was determined as per GEA Niro method [29]. The difference between poured and tapped (100 times) bulk densities gave the compressibility of the powder. Particle densities were measured by a helium gas pyconometer, AccuPyc II 1340 (Micromeritics, GA, USA). The theoretical density of the solid components of the powder, interstitial air and occluded air was calculated as per Niro (2012).

### 2.3. Flowability

Flowability was measured by two methods: 1—the time taken for a defined volume of powder to leave a rotating drum (GEA Niro, 2012); and 2—flow function measured using a Powder Flow Tester (Brookfield Engineering Laboratories Inc., Middleboro, MA, USA). For the drum flowability method, it was noticed some powder would always adhere to the inner surfaces of the drum and would not exit. Therefore, the flowability from this method was defined as a flow-rate given by: F_d_ = (g_p1_ – g_p2_)/time, where F_d_ is the drum flowability (g/s); g_p1_ and g_p2_ represent the amount of powder (g) in the drum at the start and finish of the test, respectively. Flow function was determined as described by Crowley et al. [30], who also gives an extensive description of the theory behind the measurement. Five uniaxial normal stresses (0.3 to 2.4 kPa) were applied to each powder, in combination with three over-consolidation stresses at each normal stress. The inverse of the flow function slope, also called Jenike flow index (i), was used to characterise the flow of the powders (Table 2; [15]). Compressibility was also calculated by the Powder Flow Tester by measuring the reduction in powder volume resulting from the applied uniaxial normal stresses.

### 2.4. Rehydration Properties

Powders were rehydrated to 12.5% *w*/*w* at 40 °C. Powders were added to pre-heated distilled water (120 mL) in glass baby bottles, after which the bottle was continuously inverted for 10 s.

Emulsion particle size was measured by a Mastersizer 3000 (Malvern Instruments Ltd., Malvern, UK). Particle and dispersant refractive indices were 1.46 and 1.33, respectively [7]. Particle absorption index was 0.001. Residuals (weighted and normal) were below 1% for each determination, all of which were deemed to be of good quality by the software’s internal quality check.

Viscosity was measured for each IF powder at 12.5% *w*/*w* using an AR G2 Rheometer with concentric cylinders geometry (TA Instruments, Crawley, UK). For both concentrations, samples were pre-sheared at 500 s^−1^ for 1 min followed by equilibration at 0 s^−1^ for 1 min. Shear rate was then increased from 5 to 500 s^−1^ over 2 min, held at 500 s^−1^ for 1 min, then decreased from 500 to 5 s^−1^ over 2 min.

Wettability was defined as the time taken for a given amount of powder to sink beneath the surface of 200 mL of water. The amount of powder was calculated, taking into account moisture content, to give a DM content of 12.5% *w*/*w*.

Stability of reconstituted powders to creaming and sedimentation was measured using a LumiFuge 116 stability analyser (L.U.M GmbH, Berlin, Germany). Samples were centrifuged at 1140× *g* for 3.6 h, which simulated approximately 6 months storage under normal gravity conditions. Separation behaviour was analysed using Sepview 4.1 (L.U.M GmbH, Berlin, Germany) software.

### 2.5. Statistical Analysis

Data sets were checked for normality using the Anderson Darling test. The majority of data sets were non-normal; therefore, the Kruskal Wallis test was used as a one-way analysis of variance to test the effect of single factors. Post-hoc testing was carried out using Dunn’s test [31] to determine significance between groups. Principle component analysis was performed, and powders were segregated based on powder type (I, II or III) and stage (from birth, 6 months +, 1 year +, hydrolysed). For the purposes of PCA, creaming rate values for Type III were assigned a hypothetical value of 10 mm day–1; it was not possible to measure the high rate of creaming in these samples, therefore a value was estimated to allow representation on the PCA. The statistical analysis listed above was performed using Minitab 17 (Minitab LLC, State College, PA, USA). Finally, Pearson’s r was used to determine the degree of correlation between any two sample sets with at least 6 data points i.e., Type I vs. Type II. This was calculated using the CORREL function of Microsoft Excel. All measurements were performed in at least duplicate on a single batch of each powder.

## 3. Results and Discussion

### 3.1. Powder Properties

**Figure 1:**
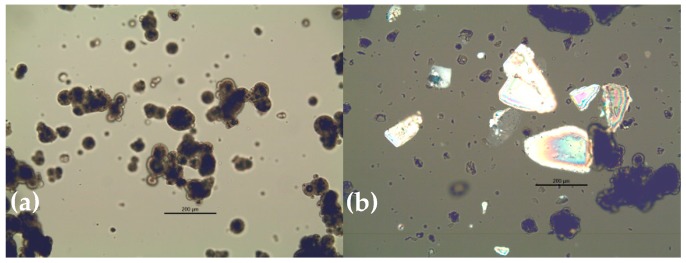
Polarised light images of (**a**) Type I (powder 1) and (**b**) II (powder 10) powders. Scale bar is 200 μm.

**Figure 2:**
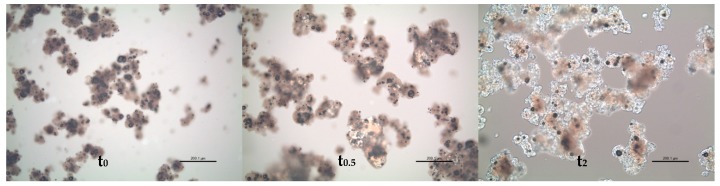
Crystallisation of powder 2 from before exposure (**t_0_**) to ambient conditions and after 0.5 days (**t_0.5_**) and 2 days (**t_2_**) of exposure.

Polarised light microscopy revealed that standard IF powders (made from intact bovine proteins) could be classified into two groups; I—homogenous powders, and II—heterogeneous mixtures of non-crystalline particles and distinct crystalline particles (Figure 1). Type I powders showed small degrees of crystallinity indicated by bright areas within particles. This may be a result of partial lactose crystallisation which could occur, for example, if powder is not cooled sufficiently after spray-drying or absorbs moisture locally during storage. Reported glass transitions for model IF powders vary in the range of 50 to 70 °C [11,24,32], and while powder temperature directly post-drying may be in this range, manufacturers generally employ cooling fluidised beds to limit crystallisation by reducing powder temperature to <30 °C before storage. The crystalline particles observed in Type II powders were very likely α-lactose monohydrate due to their pyramidal or tomahawk shape [33]. The presence of crystalline lactose in Type II powders indicated a manufacturing process where a base powder containing protein and fat ingredients was manufactured by spray-drying, after which α-lactose monohydrate crystals were added by dry-blending [3]. It is also possible that undissolved lactose crystals could be present in the concentrate prior to spray-drying. It is unlikely that these crystals could have grown during storage; Figure 2 shows that crystallisation in a Type I powder stored at ambient conditions did not result in the formation of large lactose crystals. Powders made with hydrolysed proteins were considered as a separate group (Type III). For the two Type III powders (see Table 1) containing starch, particles with the characteristic Maltese crosses, typical of starch granules, were not observed in powders. Type III powders, no. 13 and 14, contained some lactose crystals.

**Figure 3:**
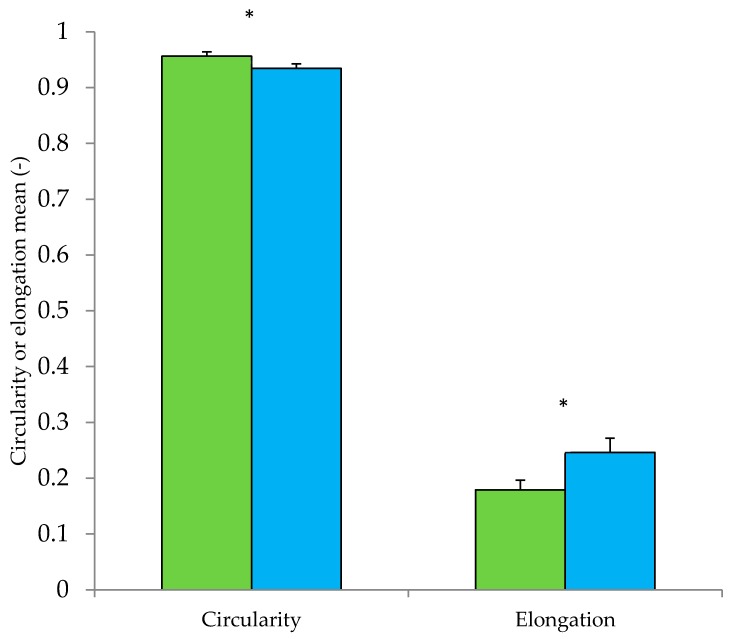
Circularity and elongation mean of Type I (**green fill**) and Type II (**blue fill**) powders. * denotes significant difference (*p* < 0.05) between columns. Note: Type I powders were made from intact proteins and did not contain large lactose crystals; Type II powders were made from intact proteins and contained large lactose crystals.

There were significant (*p* < 0.05) differences in powder particle size and shape between Type I and II powders (Table 3; Figure 3). Type I powders had fewer fine particles (D (v, 0.1)), and as a result Sauter Mean Diameter, D [3,2], was significantly larger (*p* < 0.05). D [3,2] gives the diameter of a sphere with the same volume to surface area ratio as the whole powder distribution [2]. The significantly larger D [3,2] of Type I powders, compared to Type II powders, indicated that the specific surface area of Type I powders was lower. Powder particle size was also estimated during shape analysis; similar to size analysis by laser diffraction, Type I powders were observed to have significantly (*p* < 0.05) larger particles (D [3,2] = 178.8 ± 22.9 μm) compared to Type II powders (152.3 ± 7.2 μm). Type I powders were significantly (*p* < 0.05) more spherical and less elongated than Type II (Figure 3). The presence of crystalline lactose tomahawks/pyramids in Type II powders (Figure 1) likely contributed to the greater elongation observed. Type III powders had significantly (*p* < 0.05) more fine particles compared to Type I and II powders. Within Type III powders, similar trends were observed to those observed when comparing Type I and Type II—powder 13 and 14 contained lactose crystals and had lower particle size and greater elongation than powder 15 which did not contain lactose crystals.

Surface free fat content did not vary significantly (*p* > 0.05) between Type I (0.93 ± 0.30 g free fat 100 g^−1^ powder) and Type II (0.81 ± 0.22 g free fat 100 g^−1^ powder) powders. Surface free fat of Type III powders, made using hydrolysed whey protein, was significantly (*p* > 0.05) higher than non-hydrolysed powder and varied from 1.21 to 2.15 g free fat 100 g^−1^ powder. The degree to which fat in powders was stabilised by intact milk proteins or whey hydrolysates prior to spray-drying could have affected surface free fat in powders [34]. The emulsifying ability of whey protein hydrolysates relative to intact whey proteins varies with degree of hydrolysis [35] which may have contributed to the higher, variable surface free fat content in Type III powders. Protein-to-fat ratio increased as IF progressed from formulae intended for new-borns to formulae for older babies (6 months +, 1 year +); however, in contrast to the findings of Hanley et al. [25], surface free fat did not decrease at higher protein to fat ratios. It is postulated that free fat is not only a function of composition but is also affected by processing conditions employed by the various manufacturers i.e., feed concentration, temperature profile during spray-drying, etc. [4,5].

A large degree of variability was observed within the various powder types e.g., powder 2 vs. powder 6. However, little correlation was observed between measured properties and powder stage. This was most likely due to the large number of unknown processing variables during manufacture which may affect powder properties, for example, pre-drying heat treatment [7], dryer configuration [36], breakage during transport [25], etc.

### 3.2. Flowability

Flowability of powders was measured by two means; a—Jenike flow index, and b—the rate at which powder exited from a rotating drum. Table 4 summarises the flowability data. All powders measured, with the exception of powder 15, had a flow index (i) of greater than 4 and, thus, were deemed to be easy flowing powders over the range of consolidating stresses applied [15]. There was no significant difference (*p* > 0.05) in flow index or drum flowability behaviour between Type I and II powders. Type III powders had significantly (*p* > 0.05) lower flow indices and drum flowability rates, indicative of poorer flow characteristics. Flow index and drum flowability were highly correlated for Type I powders (r = 0.95) and somewhat correlated for Type II powders (r = 0.69).

**Figure 4:**
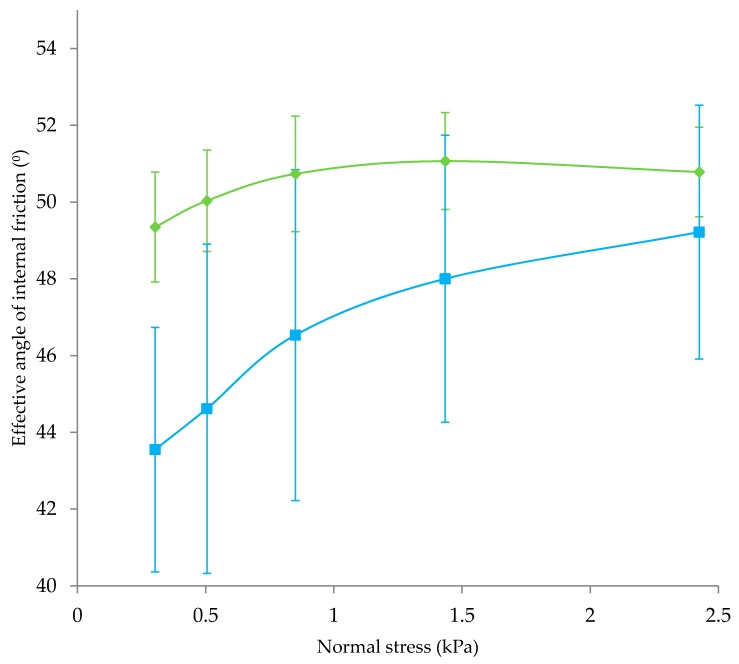
Effect of normal stress on effective angle of internal friction during flow index testing. (♦) Type I powders; (■) Type II powders. Note: Type I powders were made from intact proteins and did not contain large lactose crystals; Type II powders were made from intact proteins and contained large lactose crystals.

Type II powders had lower particle size (*p* < 0.05), were less spherical (*p* < 0.05), and were more elongated (*p* < 0.05) compared to Type I powders (Figure 3). Taking this into account, it is perhaps surprising that average flowability of Type I and II powders was not significantly different (Table 4; *p* > 0.05 for both flow index and drum flowability). Large particle size is generally desirable for good powder flowability [37]. Large powder particles reduce specific surface area compared to smaller particles, which reduces cohesive inter-particular interactions. In addition, increased spherocity of particles has been found to positively affect flowability [18]. However, particle size can often be weakly correlated with flowability [15], possibly due to the effect of surface composition, which also plays an important role in flowability [16]. Presence of distinct α-lactose crystals in Type II powders likely resulted in different overall surface composition compared to Type I powders. This difference could explain the good flow behaviour of Type II powders. Figure 4 shows the effective angle of internal friction during flow index testing. Effective angle of internal friction was lower in Type II powders, especially at lower normal stresses, indicating less resistance to flow as powder particles came into contact. At low normal forces, lactose crystals may reduce friction between particles by acting as barriers between milk powder particles containing fat at the surface. Contact between adjacent lactose crystals may also have a lower associated friction compared to milk powder particles; Yazdanpanah and Langrish [38] found that skim milk powder particles with crystalline surfaces and amorphous cores had better flowability than fully amorphous particles. At higher normal stresses, the effective angle of internal friction increased and was similar to Type I powders. This may be due to the elongated shape of lactose crystals; Fu et al. [18] found less spherical powders to have greater resistance to flow compared to more spherical particles.

Assessing powder Type I or Type II individually, increasing particle size tended to increase flowability; Pearson’s r was 0.78 and 0.73 for Type I and II powders, respectively. However, there were some differences in flowability which could not be explained by particle size. For example, Type I powders 3 and 5 had similar D [3,2], yet powder 5 was a more flowing powder. Several factors could account for the difference in flowability. Powder 5 was a more spherical powder, with a tighter size distribution, both of which could impart better flow characteristics. Surface free fat in powder 5 was lower which could also have had a positive effect, although it is hard to gauge the significance of the difference (Δ = 0.15 g free fat 100 g^−1^ powder) in this case [16]. This illustrates the complexity of powder flowability and highlights the need for multi-factorial analysis to assess flowability, even in powders with seemingly similar structures.

### 3.3. Compressibility

Compressibility was measured by two means; a—difference in powder volume before and after tapping 100 times; and b—difference in volume before and after flow index analysis. No significant (*p* < 0.05) difference in compressibility was observed between Type I and II powders. Compressibility measured during flow index testing was always higher than compressibility measured by tapping, indicating higher compressive force in the former measurement. Correlation between the two tests was high for Type I powders (r = 0.99) but for Type II powders no correlation was observed (r = 0.16). The exact reason why there was strong correlation for Type I powders and no correlation for Type II powders is unclear but may be related to the presence of lactose crystals in Type II powders.

High compressibility of powders is often reported to be related to poor flowability [18,30,39]. Table 5 shows correlation, for Type I and II powders, between all flowability and compressibility measurements. For Type I powders, compressibility was well correlated with flowability; r was between −0.9 and −1 in each case. The negative sign of r indicated that as compressibility increased, flowability decreased. For Type II powders, compressibility obtained by tapping was not correlated to flowability. Correlation of compressibility to flowability in Type II powders was higher when compressibility obtained from flow index was used. It is possible that at the lower compressive forces associated with the tapping test, lactose crystals present in Type II powders may have affected the previously reported relationship between compressibility and flowability. Yazdanpanah and Langrish [38] found that spherical particles with crystalline lactose surfaces had superior flowability to amorphous lactose, however, in Type II powders crystalline lactose had an elongated tomahawk shape (Figure 1) which can increase powder packing under compression [18]. Therefore, as the powder was compressed, both these factors could compete with each other, resulting in increased complexity compared to powders which did not contain lactose crystals. However, regardless of the exact mechanism, it is clear from Table 5 that different methods which measure the same parameter i.e., compressibility, can be influenced by powder structure. Researchers must take into account powder structure when drawing conclusions from compressibility and flowability data.

### 3.4. Rehydration to 12.5% w/w

Powders were reconstituted (12.5% *w*/*w*) in 40 °C water in a sealed glass bottle. Formulations were allowed to settle for 20 s, after which the bottles were emptied and inspected for deposits. Three of the six reconstituted Type II powders exhibited a thin deposit which had a gritty texture which was observed under the microscope to be crystalline particles with pyramidal or tomahawk shape. Deposits were not observed upon rehydration of Type I powders. Crystal sizes in the range of 200–300 μm were observed under polarised light in Type II powders, which according to Lowe and Patterson [40] could take up to 52 s to dissolve (at 37 °C) to a concentration of 100 g L^−1^. Therefore, even though the amount of lactose crystals to be dissolved during reconstitution of Type II powders was lower than 100 g L^−1^, the crystalline deposits observed were likely due to undissolved lactose crystals such as those observed in Figure 1. The exact amount of crystalline lactose in each powder was not quantified, and variations in this value may explain why deposits were not observed upon reconstitution of all Type II powders.

Reconstitution properties of powders are shown in Table 6. Wettability of powders was in the same range as previously reported [25].

Wettability time generally decreases with the presence of larger, agglomerated particles and lower free fat content [14,19,20]. In the current study, it was found that time for wettability was generally increased by larger D [3,2] (r = 0.71 and 0.68 for Type I and II powders, respectively). Powders with relatively high surface free fat were not found to be less wettable. Type II powder had similar wettability to Type I powders, despite containing a significantly larger number of fine particles of less than 100 μm (see D (v, 0.1) values in Table 3). The latter observation may be explained by the presence of lactose crystals in Type II powders, which may have made up a significant portion of the fine particles and are in general more dense, and thus sink better, than milk powder particles [29]. The apparent lack of correlation between measured variables and wettability illustrate the complexity of this widely measured parameter; particle size, surface composition (free fat), air content, etc. can all have a significant effect on wettability [41].

Apparent viscosity (500 s^−1^; 20 °C) of reconstituted powders is shown in Table 6. At 12.5% (*w*/*w*), concentration of reconstituted IF was similar to that commonly consumed by infants, and viscosity was not significantly (*p* > 0.05) different between Type I and II reconstituted powders. No consistent trend was noticed between apparent viscosity and stage of formulation (i.e., from birth to 1 year +). As the stage increases, various compositional factors changed; in general, protein content increased, casein-to-whey ratio increased, and fat content decreased. While increasing protein content and casein-to-whey ratio would generally lead to a more voluminous dispersed phase and hence higher viscosity, decreasing fat content could lead to a reduction in voluminosity of the dispersed phase [42,43]. Whey proteins are very sensitive to heat induced increases in voluminosity and also interact with casein micelles during heat treatment which could affect viscosity [44,45]. It is likely that the apparent viscosities observed were functions of all the properties mentioned above. Reconstituted Type III powders no. 13 and 14 had much higher viscosities compared to all other reconstituted powders, most likely due to the presence of starch. Powders were reconstituted at 40 °C; as starch granules do not normally start to swell and increase in viscosity until closer to 60 °C (dependant on the type of starch), this indicated that starch in powders was likely pre-gelatinised [46,47].

Particles present in Type I and II emulsions were generally smaller than 1 μm (with the exception of powder no. 3; see Table 6). Volume mean diameter D [4,3] was used for characterisation of emulsions as it is more sensitive to the presence of large particles. Higher D [4,3] in powder 3 may have been a result of relatively poor homogenisation procedures and/or destabilisation of the emulsion during manufacture. Creaming rate data, however, indicated adequate stabilisation of the fat droplets for all Type I and II powders (Table 6). The creaming rate, as measured over 6 months of accelerated storage using an analytical centrifuge, was low especially taking into account that reconstituted IF is generally consumed within a short period (less than 2 h) after preparation; the maximum creaming rate was obtained for powder no. 1 and corresponds to a movement of 29 μm over the course of 2 h. The data presented in the current study is within the same range as reported by McCarthy, Gee, Hickey, Kelly, O’Mahony, and Fenelon [6]. Creaming is generally influenced by fat droplet size and viscosity, however, no correlation was observed between these factors, likely due to differences in composition between powders; ionic strength, protein adsorbed at the oil–water interface, and pH varied throughout the samples studied, and it is hypothesised that the creaming behaviour observed was influenced, to some extent, by all these factors [48].

**Figure 5:**
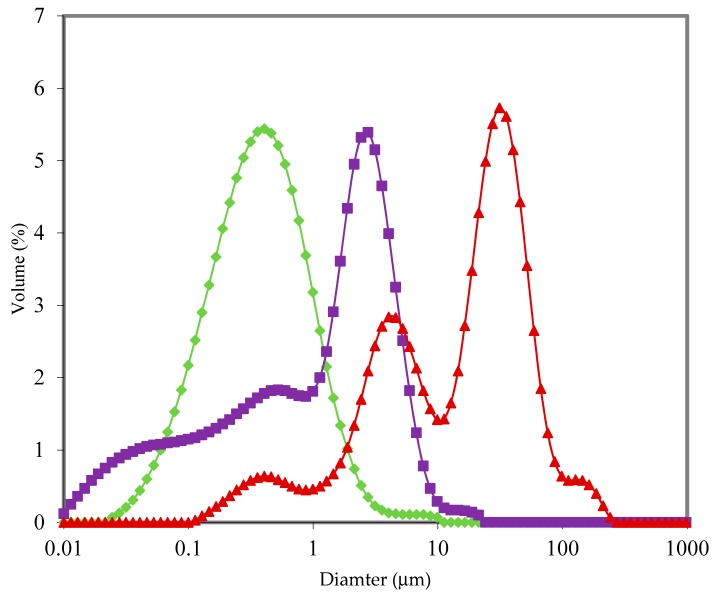
Emulsion particle size distributions of reconstituted powders (12.5%, *w*/*w*). (♦) Powder no. 2 (Type I); (▲) Powder no. 13 (Type III; starch present); (■) Powder no. 15 (Type III; no starch). Note: Type I powders were made from intact proteins and did not contain large lactose crystals; Type II powders were made from intact proteins and contained large lactose crystals; Type III powders were made from hydrolysed proteins with some powders containing large lactose crystals and starch.

Reconstituted Type III powders had much higher D [4,3] compared to Type I and II powders. Figure 5 shows size distributions of powder 2 (Type I), powder 13 (Type III; starch present), and powder 15 (Type III; no starch). For powder 13 (and 14), a large peak was observed between 10 and 100 μm, which may have corresponded to swollen starch granules. Emulsion particle size of powder 15 was not mono-modal like Type I and II powders, indicative of poorer emulsification. This is likely related to the extent of hydrolysis of the whey protein ingredient used during manufacture of powder 15 [35]. Type III powders separated more rapidly than Type I or Type II. Even though creaming rate in Type I and II powders was not deemed to be dependent on emulsion particle size, the instability of powder 15 is likely a result of much larger particles present; emulsion D [4,3] of powder 15 was over 2.5 times greater than the average of Type I and II reconstituted powders. The main destabilising mechanism observed was creaming, however, powders 14 and 15 showed significant sedimentation behaviour, likely a result of the large 10–100 μm particles shown in Figure 5. The instability of Type III powders is best illustrated by the change in transmission of light through samples over the first hour of analysis. Transmission of light through reconstituted Type III powders increased by 23.9 ± 2.6% h^−1^ compared to 3.6 ± 1.8% h^−1^ and 2.9 ± 2.0% h^−1^ for Type I and II, respectively.

### 3.5. Principle Component Analysis (PCA)

**Figure 6:**
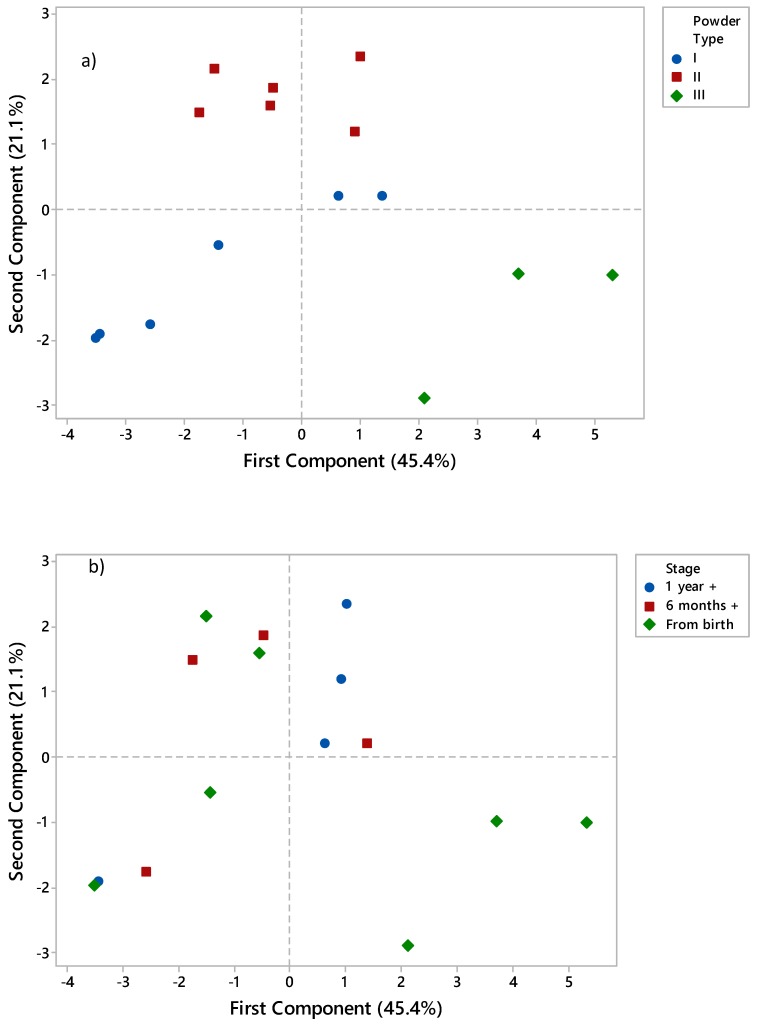
Principle Component Analysis score plots of measured variables segregated based on (**a**) powder type and (**b**) powder stage, i.e., the intended age of the consuming infant. Percentage values noted in the axes relate to the proportion of variability which is described by the respective principle component. Note: Type I powders were made from intact proteins and did not contain large lactose crystals; Type II powders were made from intact proteins and contained large lactose crystals; Type III powders were made from hydrolysed proteins with some powders containing large lactose crystals and starch.

PCA was employed to determine grouping of powders based on a transformation of the measured variables into principle components. Figure 6 shows the relationship between the first two principle components as a function of both powder type (Figure 6a) and stage (Figure 6b). Figure 6a clearly shows differentiation based on powder type. The observed differentiation between Type III and the remaining powders is not surprising given the large differences in viscosity, emulsion particle size, and creaming rate evident in Table 6. This is illustrated by the locations of the aforementioned variables in the PCA loading plot (data not shown), which are also situated in the bottom right-hand quadrant. A certain degree of separation was also possible between Type I and II powders, potentially as a result of differences in powder particle size. Type I powders exhibited larger particle size—a variable associated with the bottom left-hand quadrant. It should also be noted that differentiation between two Type I powders (powder 2 and 3) and Type II powders was poor due to their close proximity to the cluster of Type II powders (i.e., the top right-hand quadrant). Better differentiation between Type I and II powders could be achieved by increasing the number of samples used and also by taking into account more compositional and physicochemical factors. However, in the present study it was not possible to obtain better selectivity when taking into account protein and fat contents of powders (data not shown).

Differentiation between powders was not possible based on stage (Figure 6b). This indicates the difficulty in interpreting data in the absence of information regarding composition and/or processes employed during manufacture. In terms of composition, hydrolysis of whey protein and presence of pre-gelatinised starch were found to be the key factors affecting segregation of powders. Incorporation of additional compositional information, such as protein and fat contents to PCA, did not improve differentiation (data not shown). This was most likely as a result of processing variables such as pre-drying heat treatment [7]; dryer configuration [36]; breakage during transport [25], which can affect physical attributes; and behaviour of powders in manners that dominate certain innate effects associated with composition. For example, higher protein contents in 6 month + and 1 year + formulations may be expected to produce higher viscosity upon reconstitution compared to lower protein formulations; however, a severe level of heat treatment, if applied to lower protein formulations, may result in more aggregation of proteins and flocculation of fat droplets and hence, higher relative viscosity. In the current study, it seems only the relatively large compositional effects were significant enough to dominate physical properties of powders, i.e., poor emulsification properties in hydrolysed powders and high viscosity in powders containing pre-gelatinised starch.

## 4. Conclusions

The current study showed that, as hypothesised, significant variation is present in the physical properties and behaviour of commercial IF and FO products. Interpretation of the data as a function of powder structure and/or composition yielded the most interesting observations. For example, structural analysis of IF powders using the relatively simple technique of polarised light microscopy gave important information regarding the powder structure, which was instructive for interpretation of data, as well as providing interesting information on the process used during manufacture. The presence of large lactose tomahawk crystals in IF powders indicated a manufacturing process where at least some degree of dry-blending of lactose was utilised. Physical behaviour of these powders was different to powders manufactured without dry-blending of lactose and, furthermore, presence of α-lactose monohydrate crystals resulted in behaviour which was not in keeping with often reported correlations between flowability, compressibility, and particle size. With regards to composition, hydrolysis of the protein fraction was found to significantly affect the reconstituted behaviour of powders. Emulsification in hydrolysed formulations was inferior to formulations with intact proteins, indicating emulsion stability could be problematic during manufacture of hydrolysed IF.

Overall, the results presented in this study provide a useful framework for IF researchers and product developers. The ranges of physical qualities present in IF and FO products were presented, and their effect on analysis and interpretation of results was demonstrated. In particular, the importance of contextualising bulk powder and reconstituted properties as a function of powder structure and/or composition was highlighted.

## Figures and Tables

**Table 1:** Information on powders used in study.

Powder No.	Stage	Comment on Formulation */Structure **	Powder Type
1	From birth	Intact milk protein source; no lactose crystals	I
2	6 months +	Intact milk protein source; no lactose crystals	I
3	1 year +	Intact milk protein source; no lactose crystals	I
4	From birth	Intact milk protein source; no lactose crystals	I
5	6 months +	Intact milk protein source; no lactose crystals	I
6	1 year +	Intact milk protein source; no lactose crystals	I
7	From birth	Intact milk protein source; lactose crystals	II
8	6 months +	Intact milk protein source; lactose crystals	II
9	1 year +	Intact milk protein source; lactose crystals	II
10	From birth	Intact milk protein source; lactose crystals	II
11	6 months +	Intact milk protein source; lactose crystals	II
12	1 year +	Intact milk protein source; lactose crystals	II
13	From birth	Hydrolysed whey protein source; lactose crystals; starch	III
14	From birth	Hydrolysed whey protein source; lactose crystals; starch	III
15	From birth	Hydrolysed whey protein source; no lactose crystals	III

* details relating to protein source and presence of starch taken from product packaging. ** details relating to presence of lactose crystals determined by light microscopy.

**Table 2:** Jenike flow index (i) classification.

Flowability	Hardened	Very Cohesive	Cohesive	Easy-Flow	Free-Flowing
i	<1	<2	<4	<10	>10

**Table 3:** Powder particle size and surface free fat for individual powders (mean of two replicates). Average values presented below were calculated based on powder type ± standard deviation.

Powder Type	Powder No.	D [3,2] (μm)	D (v, 0.1) (μm)	Span of Particles	Surface Free Fat (% *w*/*w* of Powder)
I	1	183.0 ± 6.0	103.3 ± 1.5	1.5 ± 0.0	1.5 ± 0.0
I	2	139.0 ± 0.1	77.4 ± 0.1	1.5 ± 0.0	0.6 ± 0.0
I	3	166.2 ± 0.1	90.8 ± 0.3	1.6 ± 0.0	1.1 ± 0.0
I	4	181.4 ± 7.1	129.0 ± 3.8	1.1 ± 0.0	0.8 ± 0.0
I	5	170.0 ± 0.1	112.0 ± 0.6	1.3 ± 0.0	1.0 ± 0.0
I	6	220.1 ± 10.1	140.5 ± 6.93	1.2 ± 0.0	0.8 ± 0.0
**Average I**		**176.5 ± 25.3 ^a^**	**108.8 ± 22.3 ^a^**	**1.4 ± 0.2 ^a^**	**1.0 ± 0.3 ^a^**
II	7	160.0 ± 7.5	92.4 ± 4.1	1.6 ± 0.0	0.9 ± 0.0
II	8	162.0 ± 1.7	100.9 ± 2.1	1.5 ± 0.0	0.9 ± 0.0
II	9	117.0 ± 0.1	62.1 ± 0.1	1.8 ± 0.0	0.4 ± 0.0
II	10	149.3 ± 1.5	87.6 ± 0.7	1.6 ± 0.0	1.1 ± 0.0
II	11	143.0 ± 1.4	82.3 ± 0.1	1.6 ± 0.0	0.8 ± 0.0
II	12	126.1 ± 1.7	68 ± 0.8	1.8 ± 0.0	0.7 ± 0.0
**Average II**		**142.9 ± 17.3 ^b^**	**82.2 ± 14.0 ^b^**	**1.7 ± 0.1 ^b^**	**0.8 ± 0.2 ^a^**
III	13	125.5 ± 0.7	67.2 ± 0.2	1.6 ± 0.0	1.3 ± 0.1
III	14	112.3 ± 0.6	59.6 ± 0.2	1.7 ± 0.0	1.2 ± 0.1
III	15	134.3 ± 0.6	73.3 ± 0.3	1.5 ± 0.0	2.2 ± 0.1
**Average III**		**124.1 ± 9.6 ^b^**	**66.7 ± 5.9 ^c^**	**1.6 ± 0.1 ^b^**	**1.5 ± 0.5 ^b^**

^a,b,c^ Within a column, average values with different superscripts vary significantly (*p* < 0.05).

**Table 4:** Flowability of powders (mean of two replicates ± standard deviation). Average values presented below were calculated based on powder type ± standard deviation.

Powder Type	Powder No.	Flow Index (i)	Drum Flow (g min^−1^)
I	1	6.5 ± 0.3	32.9 ± 1.0
I	2	4.6 ± 0.3	11.2 ± 0.2
I	3	4.7 ± 0.2	17.4 ± 1.7
I	4	7.7 ± 0.8	38.5 ± 1.2
I	5	7.7 ± 0.1	38.5 ± 1.5
I	6	8.7 ± 0.5	38.2 ± 1.2
**Average I**		**6.6 ± 1.7 ^a^**	**29.4 ± 11.6 ^a^**
II	7	8.7 ± 3.4	39.0 ± 0.3
II	8	8.3 ± 0.1	29.0 ± 1.3
II	9	4.4 ± 0.3	24.8 ± 0.1
II	10	6.9 ± 0.3	35.1 ± 0.1
II	11	5.6 ± 0.4	34.6 ± 1.0
II	12	4.7 ± 0.2	20.2 ± 0.4
**Average II**		**6.4 ± 2.1 ^a^**	**30.5 ± 6.8 ^a^**
III	13	4.2 ± 0.0	20.0 ± 0.9
III	14	4.5 ± 0.4	16.5 ± 0.5
III	15	3.9 ± 0.4	14.5 ± 0.3
**Average III**		**4.2 ± 0.4 ^b^**	**17.0 ± 2.5 ^b^**

^a,b^ Within a column, average values with different superscripts vary significantly (*p* < 0.05).

**Table 5:** Correlation (Pearson’s r) between compressibility and flowability measurements for Type I and II powders (*n* = 6 for each correlation).

	Type I		Type II	
	Drum Flowability	Flow Index	Drum Flowability	Flow Index
Compressibility by tapping	−0.94	−0.90	0.35	0.33
Compressibility by flow index	−0.98	−0.93	−0.88	−0.75

**Table 6:** Reconstituted properties of commercially available infant and follow-on formula powders.

Powder Type	Powder No.	Wettability (s)	Viscosity * 12.5% (*w*/*w*) (mPa s)	pH 12.5% (*w*/*w*); 40 °C	Emulsion Particle Size D [4,3] (μm)	Creaming Rate (mm day^−1^)
I	1	18.6 ± 0.6	2.22 ± 0.01	7.01	0.70 ± 0.01	0.35 ± 0.06
I	2	10.8 ± 0.3	2.37 ± 0.06	6.99	0.60 ± 0.01	0.15 ± 0.03
I	3	15.6 ± 0.6	2.47 ± 0.05	6.89	1.17 ± 0.12	0.03 ± 0.00
I	4	16.3 ± 0.9	2.33 ± 0.04	6.65	0.57 ± 0.02	0.22 ± 0.01
I	5	16.9 ± 0.5	2.33 ± 0.03	6.61	0.56 ± 0.06	0.15 ± 0.01
I	6	16.6 ± 0.8	2.31 ± 0.01	6.55	0.67 ± 0.02	0.21 ± 0.03
**Average I**		**15.7 ± 2.6 ^a^**	**2.33 ± 0.08 ^a^**	**6.78 ± 0.20 ^a^**	**0.71 ± 0.24 ^a^**	**0.18 ± 0.11 ^a^**
II	7	24.7 ± 0.6	2.21 ± 0.06	6.79	0.38 ± 0.01	0.25 ± 0.02
II	8	32.0 ± 0.8	2.29 ± 0.08	6.81	0.80 ± 0.08	0.07 ± 0.01
II	9	14.9 ± 0.5	2.30 ± 0.03	6.76	0.54 ± 0.01	0.22 ± 0.03
II	10	16.9 ± 0.3	2.36 ± 0.04	6.77	0.41 ± 0.01	0.18 ± 0.04
II	11	15.6 ± 0.7	2.30 ± 0.04	6.76	0.54 ± 0.21	0.24 ± 0.02
II	12	19.8 ± 0.1	2.61 ± 0.01	6.70	0.72 ± 0.46	0.15 ± 0.02
**Average II**		**20.7 ± 6.5 ^a^**	**2.34 ± 0.14 ^a^**	**6.77 ± 0.04 ^a^**	**0.57 ± 0.23 ^a^**	**0.19 ± 0.07 ^a^**
III	13	14.3 ± 0.2	6.36 ± 0.07	6.55	27.81 ± 3.37	**
III	14	11.5 ± 0.1	10.91 ± 0.14	6.79	42.06 ± 4.40	**
III	15	17.61 ± 0.7	2.09 ± 0.03	6.38	2.26 ± 0.14	**
**Average III**		**14.55 ± 3.1 ^a^**	**6.46 ± 3.94 ^a^**	**6.57 ± 0.21 ^a^**	**24.41 ± 17.45 ^b^**	

^a,b^ Within a column, average values with different superscripts vary significantly (*p* < 0.05). * 500 s^−1^; 20 °C. ** Type III powders separated very quickly, and profiles were not analysed in the same manner. For subsequent PCA analysis, a hypothetical value of 10 mm day^−1^ was used in order to incorporate the significantly reduced creaming stability.
